# Seasonal trends of incidence and outcomes of cardiogenic shock : findings from a large, nationwide inpatients sample with 441,696 cases

**DOI:** 10.1186/s13054-021-03656-9

**Published:** 2021-09-06

**Authors:** Peter Moritz Becher, Benedikt Schrage, Alina Goßling, Nina Fluschnik, Moritz Seiffert, Alexander M. Bernhardt, Hermann Reichenspurner, Paulus Kirchhof, Stefan Blankenberg, Dirk Westermann, Collaboration Study Group, Alexander M. Bernhardt, Hermann Reichenspurner, Paulus Kirchhof, Stefan Blankenberg

**Affiliations:** 1 Department of Cardiology, University Heart and Vascular Center Hamburg, Hamburg, Germany; 2 German Centre for Cardiovascular Research (DZHK), Partner Site Hamburg/Lübeck/Kiel, Hamburg, Germany; 3 Department of Cardiovascular Surgery, University Heart and Vascular Center Hamburg, Hamburg, Germany

**Keywords:** Cardiogenic shock, Seasonal trends, Winter, Myocardial infarction, Mechanical circulatory support, Outcomes, Mortality

## Research letter

An increase in the annual incidence of cardiogenic shock (CS) and a growing sub-population of patients without acute myocardial infarction (AMI) was documented in Germany [1]. However, contemporary data regarding seasonal trends of CS irrespective of the underlying cause are rare.

In this study, we aimed to analyze seasonal trends of (i) incidence; (ii) patient characteristics; and (iii) outcomes in a nation-wide sample of more than 400,000 CS cases between 2005 and 2017 in Germany.

For the present analyses, all CS cases (ICD-10-GM code R57.0) in patients ≥ 18 years between 2005 and 2017 in Germany were included. Patients were categorized based on admission in one of four groups: spring, summer, fall, and winter.

**Fig. 1:**
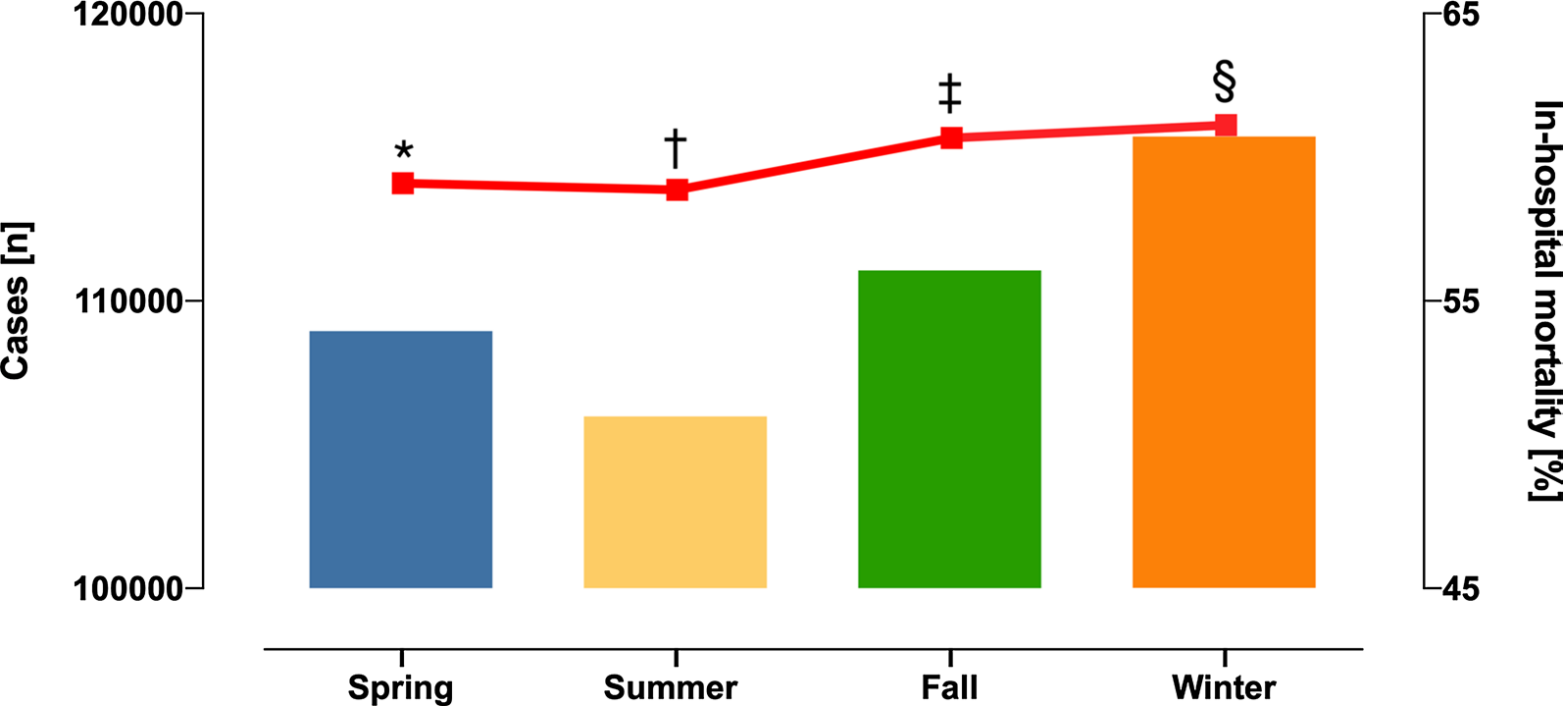
Overall seasonal trends of CS cases and in-hospital mortality from 2005 to 2017 in Germany. Seasonal variation in absolute case numbers of CS and in-hospital mortality rates (red line) over the seasons. Seasonal differences of in-hospital mortality: *p < 0.05 = Spring vs. Fall vs. Winter; Spring vs. Summer not significant. ^†^p < 0.05 = Summer vs. Fall vs. Winter; Summer vs. Spring not significant. ^‡^p < 0.05 = Fall vs. Winter vs. Spring vs. Summer. ^§^p < 0.05 = Winter vs. Fall vs. Spring vs. Summer

Temperature-related morbidity and mortality is a growing public health issue. Several studies outside Germany demonstrated more fatal and nonfatal cardiovascular events in the winter than in the summer [2], but contemporary data is missing. We show in our study: the highest incidence of CS was recorded during the winter, while the lowest incidence of CS was observed in the summer. The number of patients admitted with CS in the winter exceeded those in the summer by almost 10,000 (Table 1). Our study also revealed that in-hospital mortality of CS patients was higher in the winter than in the summer (winter vs. summer, n = 70,727 (61.1%) vs. n = 62,379 (58.8%), p < 0.001) (Fig. 1). Additionally, we found that patients admitted with CS in the winter were slightly older than in those admitted in the summer (winter vs. summer, mean age 71.1 (± 13.6) vs. 70.8 (± 13.8), whereas sex did not differ over the seasons (p = 0.8). Notably, incidence of AMI, pre-hospital and in-hospital cardiac arrest among CS patients varied across seasons as well (p < 0.001). This is in line with previous studies showing increased incidence of sudden cardiac death in the winter [3].

**Table 1:** Overall seasonal trends of CS cases in Germany from 2007 to 2015

	Winter (N = 115,710; 26.2%)	Spring (N = 108,949; 24.6%)	Summer (N = 105,980; 23.9%)	Fall (N = 111,057; 25.1%)	p value
*Demographics*					
Age, years	71.1 ± 13.6	70.7 ± 13.7	70.8 ± 13.8	71.0 ± 13.7	< 0.001
Sex, female	44,927 (38.8%)	42,141 (38.6%)	41,191 (38.8%)	43,124 (38.8%)	0.814
*Outcomes*					
In-hospital mortality	70,727 (61.1%)	64,382 (59.0%)	62,379 (58.8%)	67,381 (60.6%)	< 0.001
*Clinical presentation*					
Acute myocardial infarction	54,780 (47.3%)	52,745 (48.4%)	50,500 (47.6%)	53,967 (48.5%)	< 0.001
Pre-hospital cardiac arrest	16,540 (14.2%)	16,272 (14.9%)	16,145 (15.2%)	16,956 (15.2%)	< 0.001
Intra-hospital cardiac arrest	44,038 (38.0%)	40,818 (37.4%)	39,705 (37.4%)	42,890 (38.6%)	< 0.001
Post cardiothoracic surgery	4060 (3.5%)	3920 (3.6%)	3842 (3.6%)	3899 (3.5%)	0.329
Severe pulmonary embolism	4629 (4.0%)	4251 (3.9%)	4374 (4.1%)	4626 (4.1%)	0.006
Acute myocarditis	452 (0.3%)	318 (0.2%)	332 (0.3%)	368 (0.3%)	< 0.001
*Treatment*					
Invasive ventilation	52,354 (45.2%)	48,356 (44.3%)	46,957 (44.3%)	49,505 (44.5%)	< 0.001
Non-invasive ventilation	13,979 (12.0%)	13,220 (12.1%)	13,083 (12.3%)	13,619 (12.2%)	0.271
Dialysis	19,412 (16.7%)	18,307 (16.8%)	17,634 (16.6%)	18,423 (16.5%)	0.459
IABP	11,812 (10.2%)	11,307 (10.3%)	10,689 (10.0%)	10,777 (9.7%)	< 0.001
LVAD	507 (0.4%)	494 (0.4%)	496 (0.4%)	468 (0.4%)	0.398
VA-ECMO	2374 (2.0%)	2453 (2.2%)	2430 (2.2%)	2568 (2.3%)	< 0.001

*IABP* intra-aortic balloon pump, *LVAD* left ventricular assist device, *VA-ECMO* veno-arterial extracorporeal membrane oxygenation

The field of temporary mechanical circulatory support (MCS) to manage patients with CS enhanced in the last decade [4]. In this study, intra-aortic balloon pump (IABP) was the most used assist device, followed by veno-arterial extracorporeal membrane oxygenation (VA-ECMO) and left ventricular assist device (LVAD) in CS patients, illustrating the perceived clinical need for MCS devices.

The multidisciplinary shock team approach utilizing protocol-driven care appears to be feasible and to reduce mortality in patients with refractory CS [5, 6]. However, the extent to which the shock team approach and associated outcomes are affected by seasonal variations remains unclear. Further studies have to elucidate whether prolonged transport time due to adverse weather conditions, atherosclerotic/thrombotic incidences in terms of AMI, and time-dependent care processes are influenced by seasonal variations and/or lower temperatures.

The strengths of this study are the large sample size and the well-validated database. Clinical variables such as laboratory values, physiological markers and follow-up data beyond the hospital stay were unfortunately not available in this administrative dataset. The exact time course of the different diagnoses e.g. being prevalent at admission or incident during the hospital stay was not possible to assess in this administrative dataset. This potential bias/confounding has to be taken under consideration when interpreting our results. Finally, validation of our results outside of Germany is needed.

In this nation-wide cohort of more than 400,000 CS patients, incidence and in-hospital mortality of CS varied substantially by season, with lowest incidence/mortality during the summer and highest incidence/mortality during the winter. A better understanding of these seasonal trends, and especially if these can be attributed to temperature changes or factors related to quality of care, needs to be evaluated in future research. This might have important implications for the care of CS patients and could help to improve outcomes.

## Data Availability

Data and material are available.

## Code availability

Software codes are available.
